# Evaluations of dyadic synchrony: observers’ traits influence estimation and enjoyment of synchrony in mirror-game movements

**DOI:** 10.1038/s41598-024-53191-0

**Published:** 2024-02-05

**Authors:** Ryssa Moffat, Emily S. Cross

**Affiliations:** 1 Professorship for Social Brain Sciences, ETH Zurich, Zurich, Switzerland; 2 School of Psychological Sciences, Macquarie University, Sydney, NSW Australia; 3 MARCS Institute, Western Sydney University, Sydney, NSW Australia

**Keywords:** Human behaviour, Social neuroscience

## Abstract

While evidence abounds that motor synchrony is a powerful form of ‘social glue’ for those involved, we have yet to understand how observers perceive motor synchrony: can observers estimate the degree of synchrony accurately? Is synchrony aesthetically pleasing? In two preregistered experiments (n = 161 each), we assess how accurately observers can estimate the degree of synchrony in dyads playing the mirror game, and how much observers enjoy watching these movements. We further assess whether accuracy and enjoyment are influenced by individual differences in self-reported embodied expertise (ability to reproduce movements, body awareness, body competence), psychosocial resources (extraversion, self-esteem), or social competencies (empathy, autistic traits), while objectively controlling for the degree of measured synchrony and complexity. The data revealed that observers’ estimated synchrony with poor accuracy, showing a tendency to underestimate the level of synchrony. Accuracy for low synchrony improved with increasing body competence, while underestimation for high synchrony rose with increasing autistic traits. Observers’ enjoyment of dyadic movements correlated positively with the degree of measured synchrony, the predictability of the movements, and the observer’s empathy. Furthermore, very low enjoyment was associated with increased body perception. Our findings indicate that accuracy in perceiving synchrony is closely linked to embodiment, while aesthetic evaluations of action hinge on individual differences.

## Introduction

Motor synchrony is a form sustained coupling of movements in space and time between two or more people that emerges spontaneously in everyday situations^1^. Pedestrians fall effortlessly into matching strides, musicians in ensembles sway in concert with each other, and conversational partners even tend to perform matching eye movements^2–5^. This unpremeditated matching of body movements promotes prosocial behavior and is a form of ‘social glue’^6–9^, which signals group cohesion^10–12^, and has the potential to enhance cooperation^13,14^ and cognitive functioning^15–17^. Theoretical perspectives suggest that motor synchrony is an adaptive behaviour that aids communication and helps strengthen relationships^7,18,19^.

Given the quotidian emergence of motor synchrony, we stand high chances of engaging spontaneously in motor synchrony and also of observing others moving synchronously^20^. The question of how observers perceive synchrony has received some empirical attention, with findings demonstrating that observers can identify the presence of synchrony, but estimate the degree of synchrony with variable accuracy^21–23^. In the present study, we use ‘accuracy’ to refer to the mismatch between the level of synchrony that observers perceive and the objectively measured level of synchrony. Recent work has demonstrated that observers’ enjoyment (or aesthetic appreciation) of shared movement correlated positively with the degree of motor synchrony between the moving individuals, but that observers were not cognizant of the degree of motor synchrony that they observed^23,24^. While we expect that this reported mismatch between perceived and objectively measured motor synchrony would replicate in a larger sample, it is also plausible that some individuals judge synchrony with greater accuracy than others, but that this information is diluted or lost in group-level analyses. In this preregistered study, we examine the relationship between the accuracy with which lay observers estimate the degree of motor synchrony in short videos of dyads playing the mirror game, i.e., one person moving their arms spontaneously and freely while the other tries to match the movements as closely as possible in time and space^15,16,25^. We also examine observers’ enjoyment of the movements, movement complexity, as well as specific interindividual traits. In doing so, we offer future studies a springboard from which to delve further into individual differences in the perception and aesthetic appreciation of motor synchrony, a phenomenon that likely has implications for understanding individual differences in social behavior^18,26^.

### Estimating the degree of motor synchrony … accurately

Over the past four decades, researchers have queried observers’ perceptions of synchrony without verifying their accuracy. In an early study probing observers’ sensitivity to synchrony, observers’ watched real interactions of two people, and interactions of two people spliced together, and rated the real interactions as more synchronous than the spliced interactions^27^. Vacharkulksemsuk & Fredrickson^28^ explored whether observers’ ratings of the degree of synchrony within a dyad might mediate the relationship between self-disclosure and embodied rapport. Observers’ ratings have also been associated with the spatiotemporal coordination and period-locking of dyad’s joint movements, suggesting that observers make use of this kinematic information^29–31^. While observers’ subjective estimation of movement synchrony has been demonstrated to help explain some social behaviors, we propose that an additional objective measure of synchrony is needed to contextualize these results^32^. In other words, how do we interpret these findings, if we do not know how accurate observers’ judgements are?

To date, only a handful of studies have compared subjective and objective measures of motor synchrony^21–23^. Most recently, in a successful replication of Lumsden et al.^21^, Macpherson et al.^22^ asked observers to watch and rate the synchrony between two hands, belonging to different people (forearms fixed on table, hands moving vertically). To obtain an objective measure of synchrony, the authors recorded and compared the angles of the dyads’ wrist-bends over time using electro-goniometers. When comparing this measure of synchrony to observers’ ratings, they found that observers’ estimations were biased by differences in skin tone, whereby observers underestimated synchrony. The authors of both studies propose that the accuracy with which observers estimate the degree of motor synchrony is susceptible to non-task-related information.

In a study examining full-body movements, Vicary et al.^23^ assessed the Granger causality of the objective and perceived motor synchrony, as well as observers’ enjoyment and heart rate, during choreographies that combined ten performers walking and waving. Motor synchrony between performers was recorded using wrist-accelerometers, and observers assessed their own enjoyment of the movements, as well as the performers’ ‘togetherness’ continuously throughout the performance. Vicary et al.’s analyses suggest that raters were poor judges of the degree of synchrony in the performances, and that raters may have mischaracterized increased acceleration and visual change as ‘togetherness’. Further, the presence of objectively measured synchrony was predictive of raters’ enjoyment and physiological arousal, but only when raters were decidedly appreciative or unappreciative of the performance. One commonality across Lumsden et al.^21^, Macpherson et al.^22^, and Vicary et al.’s^23^ studies is that observers’ accuracy lacks stability and is highly influenceable by contextual factors. Vicary et al. also suggest that differences in levels of enjoyment may predict accuracy of ratings, but Vicary et al.’s study only offers group-level insight, which may obscure important differences at the level of the individual^33^. In the present study, we aim to elucidate whether individual differences may explain differences in accuracy.

### Established perspectives on action aesthetics

Numerous studies have assessed how kinematic parameters shape aesthetic appreciation of movements made by expert dancers. Movements deemed aesthetically pleasing were greater in amplitude^34,35^, faster^35,36^, and involved prolonged balancing^35^, and also covered more distance while airborne^37^. Moreover, embodiment (or bodily familiarity with movements) has been demonstrated to influence one’s enjoyment of human body movements in two ways: first, observing complex movements beyond one’s own bodily capacities evokes appreciation and, second, heightened embodiment in expert dancers and athletes allows for greater appreciation of movements from their specific repertoire^37–41^. These findings demonstrate that kinematic properties of movement can shape aesthetic appreciation, as can one’s embodied movement expertise. Emerging evidence continues to highlight the extent to which an individual’s experiences, skills, and cultural knowledge may underpin aesthetic appreciation, and has led empirical aesthetics researchers to call for greater emphasis on a wider spectrum of individual differences in future studies^39,42–45^.

### Individual differences-embodiment and beyond

Understanding aesthetic judgements requires in-depth attention to specific individual differences^39,42–45^. This view is aligned with the meta-analytic evidence that interpersonal accuracy, that is people’s ability to perceive and interpret relevant social information^46^, depends not only on the strength of the information being transmitted, but also the individual’s sensitivity to this social information^47^. This sensitivity has been associated with individual differences in traits including extraversion, empathy, as well as social knowledge and competence^48,49^. In the present study, we consider individual differences related to embodiment, as well as socially relevant traits including extraversion, self-esteem, empathy, and autistic traits. We acknowledge that these traits are not exhaustive, and that much of the literature on these traits explores behavior from a second-person perspective, rather than the third-person perspective we will explore. It is our intention to contribute robust evidence to this topic, which can in turn be used to guide future research on the aesthetic appreciation of social interactions, beginning with dyadic synchronous movements.

#### Embodiment: reproducibility of movements, body competence, and body perception

First, we consider the contribution of embodied expertise to perceptions of synchronous movements. Research on the sensory integration of sound and movement offers insights into the role of embodiment in perceiving and maintaining synchrony. For example, expert musicians, relative to non-experts, possess embodied rhythmic knowledge that may enhance their ability to synchronize their movements with sounds or other people’s movements and to detect non-synchronous sound–movement pairings^50–54^. Accordingly, one might expect movement experts (e.g., athletes or coaches) or interpersonal communication experts (e.g., therapists or mediators) to have embodied kinematic knowledge that shapes perception of movement synchrony. In the present work, we seek to explore how differences in non-expert levels of embodiment may modulate lay observers’ estimation of synchrony, as well as their aesthetic appreciation thereof.

We follow in the steps of work on dance aesthetics by assessing how ‘reproducible’ participants find movements^41,55^. However, as we invited lay people to perform the dyadic mirror-game movements, the movements are relatively simple and ‘reproducible’ (i.e., do not require any expertise to be performed or perceived). Because of this, we also explore two measures of embodiment that tap into individual traits that may promote or hinder embodiment: body competence, the strength of one’s belief that one’s body performs physical activities competently^56^ and body perception, or how strongly one attends to one’s bodily signals^57^. These measures offer insight into contrasting facets of embodiment. Greater self-reported body competence likely reflects greater bodily expertise or greater embodiment, which may enhance perception of subtle information conveyed in body movement. For example, the number of years spent training a sport is positively correlated with observers’ abilities to estimate the intensity of emotion expression in point-light displays^58^, i.e., dots displayed on a screen representing human body parts in motion. On the other hand, perception of one’s bodily signals, when over- or underdeveloped, may reduce sensitivity to social information conveyed by others’ bodies, impacting social competence negatively^59–61^. It is thus plausible that an inward focus might interfere with embodiment, potentially altering the accuracy and enjoyment with which individuals perceive motor synchrony.

#### Psychosocial resources: extraversion and self-esteem

Next, we explore the roles of psychosocial resources, extraversion and self-esteem, which influence how observers perceive biological movement and faces^62–64^. Individuals with high extraversion scores engage in more motor synchrony during conversation^65^ and use representational gestures more frequently^66^, suggesting greater relevance of embodiment in everyday interactions. Tabak et al.^64^ report a relationship between observers’ extraversion and abilities to recognize emotions in point-light displays, which the authors suggest is likely mediated by the observers’ empathy. It may, therefore, be that observers with greater extraversion are more attuned to subtle differences in the degree of synchrony in dyadic movements and may experience greater enjoyment while viewing synchronous movements.

For individuals with low self-esteem, ostracism can reduce sensitivity to movement information in point-light displays^62^. Macpherson et al.^22^ demonstrated that individuals with greater social anxiety, which is negatively correlated with self-esteem^67,68^, showed reduced accuracy when estimating the degree of synchrony in rhythmic hand movements. Based on these very limited findings, we explore whether individual differences in self-esteem may result in differing levels of accuracy when estimating the degree of synchrony in dyadic movements, as well as possible differences in enjoyment.

#### Social competence: empathy and autistic traits

Finally, we explore the relationship between measures of social competence (empathy and autistic traits) and their influence on perceptions of synchrony. There appears to be a strong link between empathy and sensitivity to information conveyed through human body movements, in both behavior^58,69^ and brain activity^70–72^. Considering behavior, greater empathy can lead to improved recognition of emotional intensity^58^ and enhanced social bonding with others in the presence of music^69^ when observers view simplified representations of human movement, i.e., stick figures or dots. In the brain, the excitability of the motor cortex while watching another person move–or dance–correlates with the observer’s empathy score^71,72^. The inferior frontal gyri (IFG) and inferior parietal lobule (IPL) are also involved in representing observed movements, and show increased activation in observers with greater empathy^70^. Considering these studies together, we anticipate that individual differences in empathy may shape observers’ perceptions of dyadic synchrony, both in terms of assessing the similarity of movements and aesthetic appreciation.

Autistic traits, commonly associated with social difficulties^18^, are also linked with reduced sensitivity to biological movement^73–75^. This may be the result of hyperactivation of the mirror-neuron system^76^. Further, Williams & Cross^77^ demonstrated that observers with fewer autistic traits prefer human movement relative to non-human or machine-like movements and will engage in more effortful tasks to view their preferred movements, whereas observers with more autistic traits did not show this preference. The authors suggest that this preference for human movement may stem from underlying differences in the reward experience of individuals reporting differing numbers of autistic traits. More specifically, human movement may be more rewarding for observers with fewer autistic traits than observers with more autistic traits. Finally, in the only study to date to assess individual differences in accuracy when estimating the degree of dyadic synchrony, Macpherson et al.^22^ found no relationship between autistic traits and accuracy of synchrony perception. While Macpherson et al. employed videos of hands making unvarying rhythmic movements, we explore the subtleties of spontaneous body movements in the present study. With respect to perceptions of dyadic motor synchrony, we propose that observers with a greater number of autistic traits may be less sensitive to subtle details conveyed in human movements and experience less enjoyment while watching them.

### Current study

In this study, our first aim is to replicate Vicary et al.’s^23^ findings, that people estimate the degree of motor synchrony inaccurately, using a larger sample. Our second aim is to examine whether this phenomenon generalizes across individuals manifesting diverse profiles of embodiment, psychosocial traits, and social competencies. In doing so, we respond to the call to consider individuals’ aesthetic preferences in the context of their unique being^39^. Here, we present our findings from our analyses of two samples (each n = 161) separately, as preregistered, and together.

As preregistered (https://osf.io/ugczs/), we hypothesized that people will estimate the degree of synchrony in videos of dyads playing the mirror game with poor accuracy (*Hypothesis 1*). To assess this, we will subtract observers’ estimates of the degree of synchrony from the objectively measured degree of synchrony, yielding a difference value. Difference values of zero indicate high accuracy. Difference values above zero indicate underestimation and values below zero indicate overestimation of synchrony. We expect the group-level difference value to differ from zero, indicating poor accuracy in estimating the degree of observed motor synchrony. Next, to address the possibility that stimulus complexity may influence ratings^41^, we formed an exploratory hypothesis that more predictable movements may yield better accuracy (*Hypothesis 2*). We also hypothesized that enjoyment and movement reproducibility may be positively associated with the accuracy with which people estimate the degree of synchrony (*Hypothesis 3*). A further exploratory hypothesis was that specific interindividual traits may influence how accurately people estimate the degree of synchrony, in that extraversion, self-esteem, body perception, body competence, empathy, and/or autistic traits may be associated with accuracy (*Hypothesis 4*). We also examined one further hypothesis, which was not preregistered: Enjoyment of dyadic movement may be influenced by movement predictability and similarity, as well as specific interindividual traits (*Hypothesis 5*).

## Results

**Figure 1:**
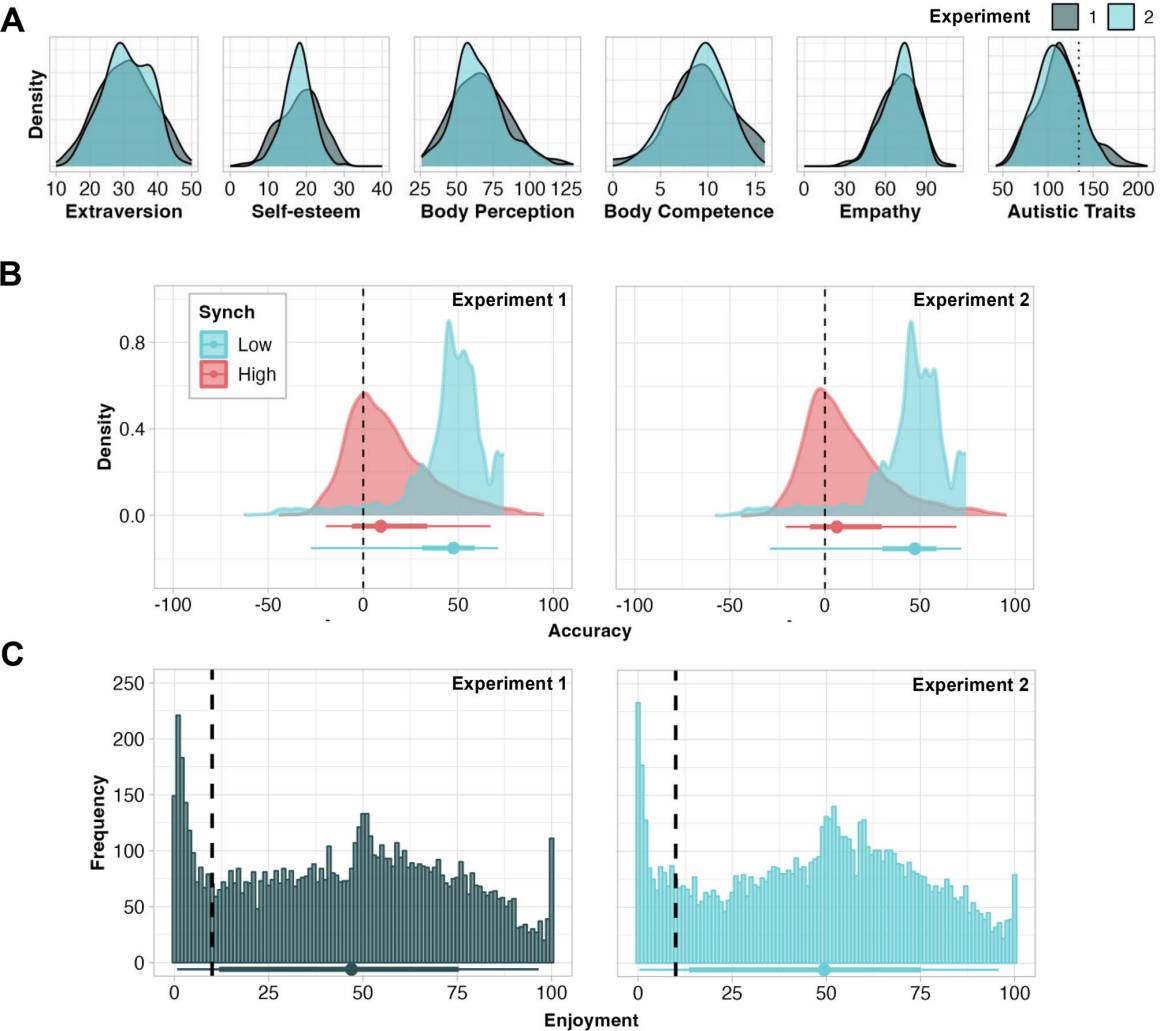
(**A**) Distribution of scores on individual measures in raw units. Density axis not shown on figure to normalize by maximal values. Dotted vertical line in autistic traits panel indicates the threshold above which scores are likely to reflect autism spectrum disorders. Each measure was z-scored prior to inclusion in models. (**B**) Distributions of accuracy (calculated by subtracting observers’ estimations of movement similarity from objectively measured movement similarity) for high and low synchrony stimuli. Positive values = underestimation; negative values = overestimation; 0, marked by dashed vertical line = no difference (i.e., a perfect estimation). Summary point shows median, and bars show interval covering 66 and 95% of the raw distribution. Parameter estimates in shown in Supplementary Table 2. (**C**) Histograms of enjoyment ratings. Summary point shows median, and bars show interval covering 66 and 95% of the raw distribution. The dashed vertical line indicates rating of 10. Ratings ≤ 10 were coded as “very low enjoyment”. Ratings 11–100 were coded as enjoyment “per se”.

As per our preregistrations, we fit Bayesian multilevel models to the data from each experiment (refer to Methods for details). We observed that some relationships varied between experiments and subsequently, we combined the data from Experiments 1 and 2 to explore how these relationships manifest in a larger sample that is plausibly more robust to sampling error. As described in greater detail the Methods, we report parameter estimates with a 95% credible interval spanning the highest posterior density region (HPD)^78^. Distributions for each of the individual difference measures are shown in Fig. 1A, with descriptive statistics presented in Supplementary Table 1 (Sect.  1 of Supplementary Material). Distributions and descriptive statistics for participants’ estimations of synchrony and their ratings of enjoyment are visualized in Fig. 1B, C.

### Observers’ accuracy in estimating the degree of synchrony in mirror-game movements

#### Observers underestimate the degree of synchrony

Our first aim was to replicate Vicary et al.’s^23^ finding that observers are poor judges of synchrony. In both experiments, overall accuracy indicated that observers underestimate synchrony (Experiment 1: β  = 28.30, HPD = [26.90, 29.70]; Experiment 2: β = 26.90, HPD = [25.40, 28.40], Experiment 1 + 2: β = 27.70, HPD = [26.60, 28.80]), with greater underestimation for low than high synchrony (Fig. 1B and Supplementary Table 2), confirming our hypothesis (1).

#### Greater movement predictability improves accuracy for low, but not high, synchrony movements

**Figure 2:**
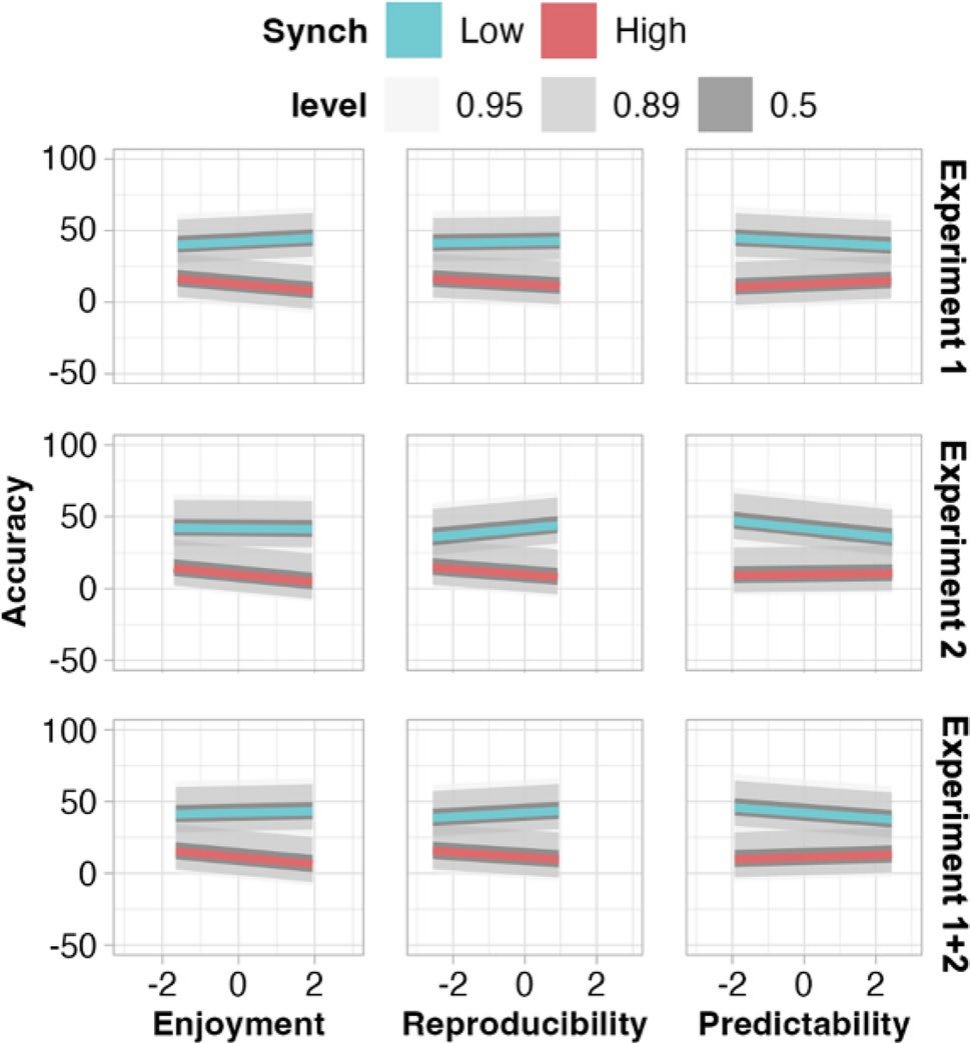
Relationship between accuracy and each enjoyment, reproducibility, and predictability of movements, per degree of synchrony and experiment. For predictability, greater positive values represent less predictability, as we calculated entropy levels, wherein smaller values represent a ‘purer’, more predictable, signal. Level shading shows interval covering the stated percentage of the posterior distribution per parameter. Parameter estimates shown in numeric form in Supplementary Table 3.

Next, we assessed whether the predictability of movements was associated with increased accuracy (Fig. 2) as per our hypothesis (2). In both experiments, greater predictability was associated with better accuracy (reduced underestimation) for low synchrony (Experiment 1: β = −1.17, HPD = [−2.37, −0.02], Experiment 2: β = −2.61, HPD = [−3.78, −1.41]). For high synchrony, no association was observed in Experiment 1 (ß = 0.32, HPD = [−0.12, 0.76]) while greater predictability corresponded with greater underestimation in Experiment 2 (β = 1.05, HPD = [0.60, 1.50]). Analysis of the aggregated sample from Experiments 1 and 2 showed that predictability of movement was associated with increased accuracy for low synchrony (−1.99, HPD = [−2.77, −1.10]) and decreased accuracy for high synchrony (β = 0.69, HPD = [0.38, 1.02]). Note: as we used entropy as a proxy for predictability, values closer to zero signify greater predictability than values further from zero.

#### Greater ratings of enjoyment and reproducibility improve accuracy for high-synchrony movements

We hypothesized (3) that greater enjoyment and reproducibility ratings would be associated with better accuracy and observed this for high-synchrony movements across both experiments (less underestimation; Fig. 2 and Supplementary Table 3). However, for low synchrony, Experiment 1 suggested that accuracy improved as enjoyment decreased, but showed no association between accuracy and reproducibility. In Experiment 2, for low synchrony, greater reproducibility were associated with reduced accuracy, whereas enjoyment was not. The combined data from both experiments demonstrated that, for high synchrony, greater enjoyment and reproducibility are associated with improved accuracy, and for low synchrony, greater reproducibility resulted in reduced accuracy for high synchrony (i.e., greater underestimation).

#### Greater body competence improves accuracy for low-synchrony movements

**Figure 3:**
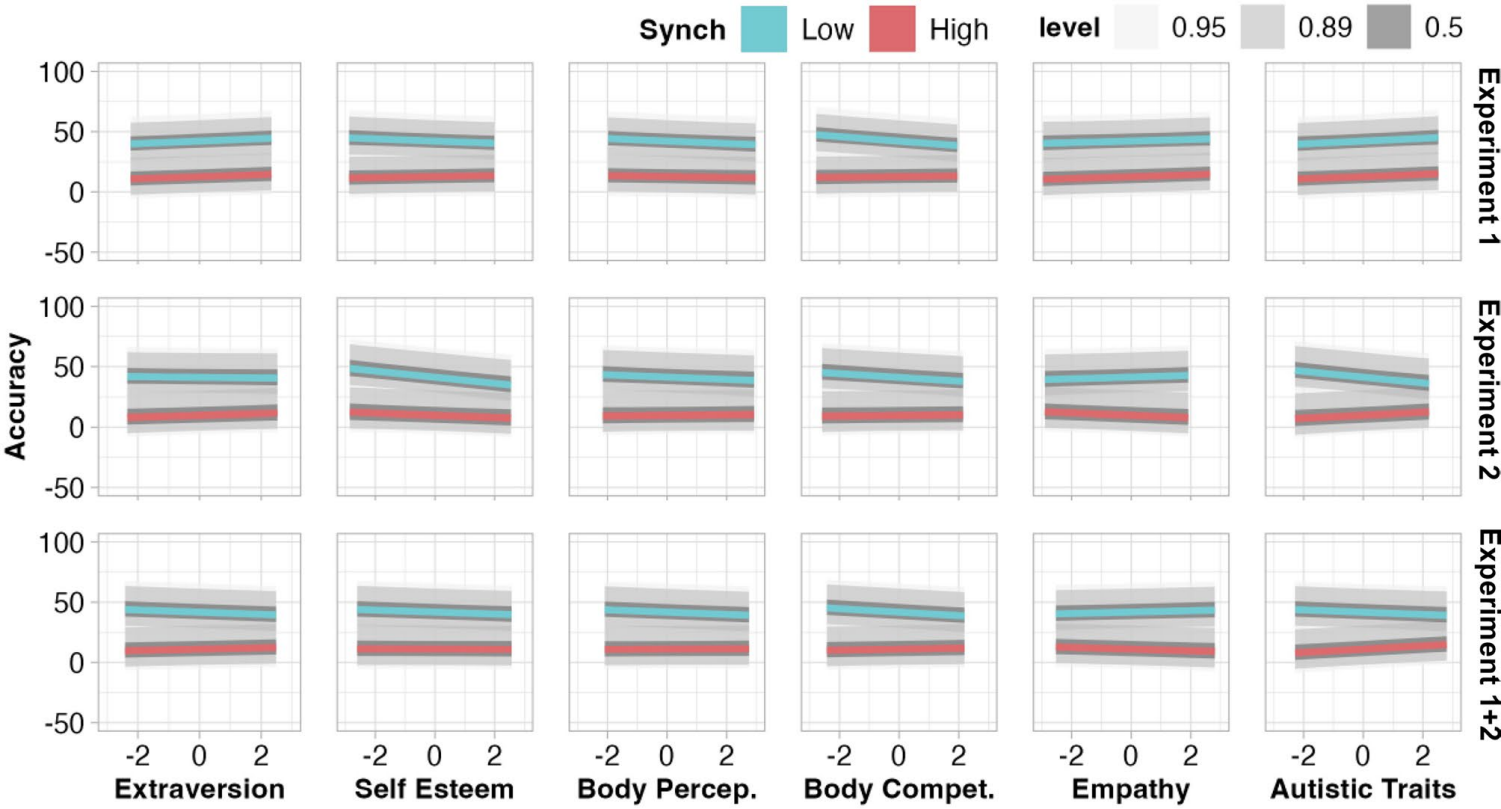
Relationship between accuracy and interindividual measures, per degree of synchrony and experiment. For predictability, greater positive values represent less predictability, as we calculated entropy levels, wherein smaller values represent a ‘purer’, more predictable, signal. Level shading shows the interval covering the stated percentage of the posterior distribution per parameter. Parameter estimates reported in Supplementary Table 4. *Body Percep.*  body perception, *Body Compet.*  body competence.

We subsequently assessed the relationship between accuracy and measures of extraversion, self-esteem, body perception, body competence, empathy, and autism traits to address our hypothesis (4) that individual traits may influence accuracy. For a high synchrony, no interindividual measures predicted accuracy in either experiment (Fig. 3 and Supplementary Table 4). For low synchrony, Experiment 1 revealed only body competence to be associated with accuracy, whereby increased body competence was associated with reduced underestimation (i.e., improved accuracy). Experiment 2 showed a matching trend for body competence (88% of HPD below 0). Further, for low synchrony, Experiment 2 showed trending associations between improved accuracy and greater self-esteem (96% of HPD below 0) as well as reduced accuracy and more autism traits (94% of HPD below 0; Supplementary Table 4). The same model, fit to the aggregated data from Experiments 1 and 2, indicated the same negative relationship between body competence and accuracy for low synchrony, as well as positive relationship between autistic traits and greater underestimation for high synchrony (Fig. 3).

### Ratings of enjoyment while observing synchrony in mirror-game movements

As illustrated in Fig. 1C, the distributions of enjoyment ratings from both experiments have two peaks: one around 50 and another at approximately 0 or 1. To model this distribution and address our exploratory hypothesis (5) that individual traits may influence enjoyment, we used zero-inflated gaussian models. We employed these to predict the likelihood of ratings close to zero (very low enjoyment, ratings ≤ 10) per parameter, as well as the relationship between the rest of the ratings (enjoyment per se, ratings 11–100) and each parameter.

#### Very low enjoyment increases with greater awareness of bodily signals

**Figure 4:**
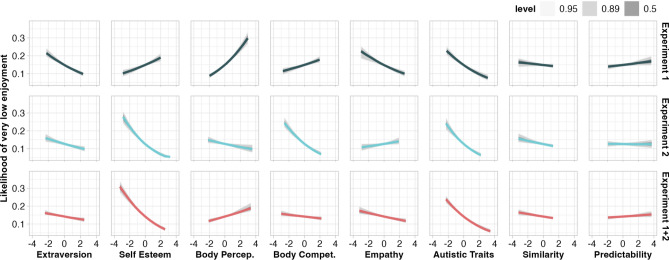
Relationship between very low enjoyment and interindividual measures, as well as measured similarity and predictability, per experiment. For predictability, greater positive values represent less predictability, as we calculated entropy levels, wherein smaller values represent a ‘purer’, more predictable, signal. Level shading shows the interval covering the stated percentage of the posterior distribution per parameter. Parameter estimates reported in Supplementary Table 5. *Body Percep.* body perception, *Body Compet.* body competence.

In Experiment 1, we found that greater extraversion, empathy, and autism traits correlated with a reduced likelihood of very low enjoyment (ratings 0–10/100; Fig. 4). Greater self-esteem, body perception, and body competence measures were associated with a greater likelihood of very low enjoyment. In Experiment 2, increased extraversion and autistic traits were associated with a reduced likelihood of very low enjoyment (consistent with Experiment 1). In direct opposition to our findings from Experiment 1, Experiment 2 showed greater self-esteem, body perception, and body competence to be correlated with a reduced likelihood of very low enjoyment, while a trend for greater empathy to be associated with a greater likelihood very low enjoyment was also observed (93% of HPD above 0; Fig. 4 and Supplementary Table 5). We were surprised to observe effects of a similar size in opposite directions (for self-esteem, body perception, body competence, and empathy) across these two experiments, and suspect these may be the result of natural variation between experiment samples. Analysis of the aggregated data from both experiments showed that greater scores on all traits were associated with a reduced likelihood of very low enjoyment, except body perception, which was positively associated with very low enjoyment (Fig. 4).

#### Greater empathy enhances enjoyment per se

**Figure 5:**
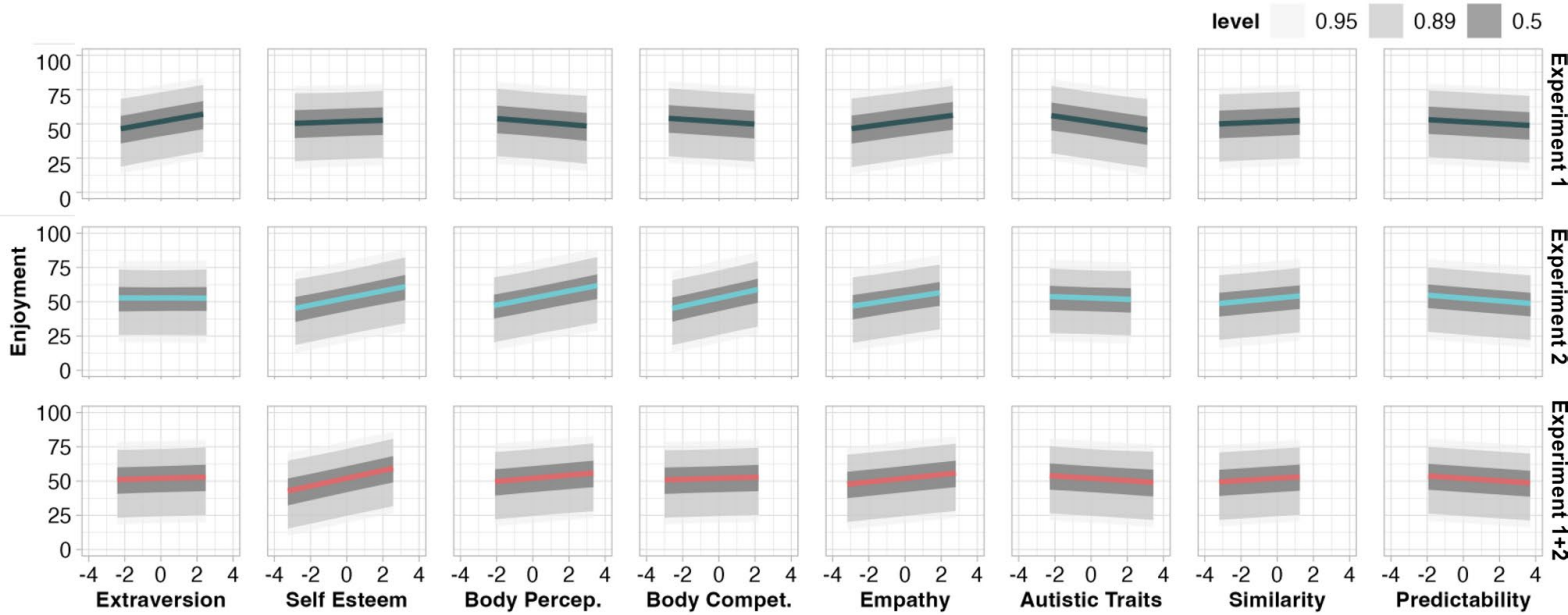
Relationship between enjoyment per se and interindividual measures, as well as measured similarity and predictability, per experiment. For predictability, greater positive values represent less predictability, as we calculated entropy levels, wherein smaller values represent a ‘purer’, more predictable, signal. Level shading shows the interval covering the stated percentage of the posterior distribution per parameter. Parameter estimates reported in Supplementary Table 6. *Body Percep.*  body perception, *Body Compet.* body competence.

In Experiment 1, enjoyment per se (ratings 11–100/100) increased with greater extraversion. We also observed trends of increasing enjoyment with greater empathy (92% of HPD above 0) and decreasing enjoyment with increasing autistic traits (93% of HPD below 0). No relationship was observed between enjoyment and self-esteem, body perception, or body competence in Experiment 1. In Experiment 2, we observed a positive association between enjoyment and empathy (consistent with Experiment 1), as well as increasing enjoyment with increasing self-esteem, body perception, body competence and enjoyment (not observed in Experiment 1). No relationship was observed between enjoyment and extraversion or autistic traits in Experiment 2 (Fig. 5 and Supplementary Table 6). For Experiments 1 and 2 together, greater empathy correlated with greater enjoyment, and the remaining trait–enjoyment relationships observed in Experiments 1 and 2 dissipated.

#### Greater movement predictability may increase likelihood of very low enjoyment

In Experiment 1, we observed a trend in which lower predictability was associated with very low levels of enjoyment (92% of HPD above 0), which was not replicated in Experiment 2 (Fig. 4). Enjoyment per se was not associated with predictability in Experiment 1 but rather positively associated in Experiment 2. Analysis of the aggregated data showed no evidence for a relationship between predictability and very low enjoyment, and that greater predictability was associated with greater enjoyment per se (Fig. 5; Supplementary Tables 5 and 6).

#### Greater movement similarity enhances enjoyment and reduces likelihood of very low enjoyment

Experiment 1 and 2 revealed greater measured similarity of movements to be associated with increased enjoyment per se (Fig. 5). Experiment 1 did not show similarity to be associated with the likelihood of very low enjoyment, whereas Experiment 2 suggested that greater measured similarity reduced the likelihood of very low enjoyment (Fig. 4). Analysis of the aggregated data from both experiments showed the same pattern as Experiment 2 for both very low enjoyment and enjoyment per se (Fig. 4 and Fig. 5; Supplementary Tables 5 and 6).

## Discussion

**Figure 6:**
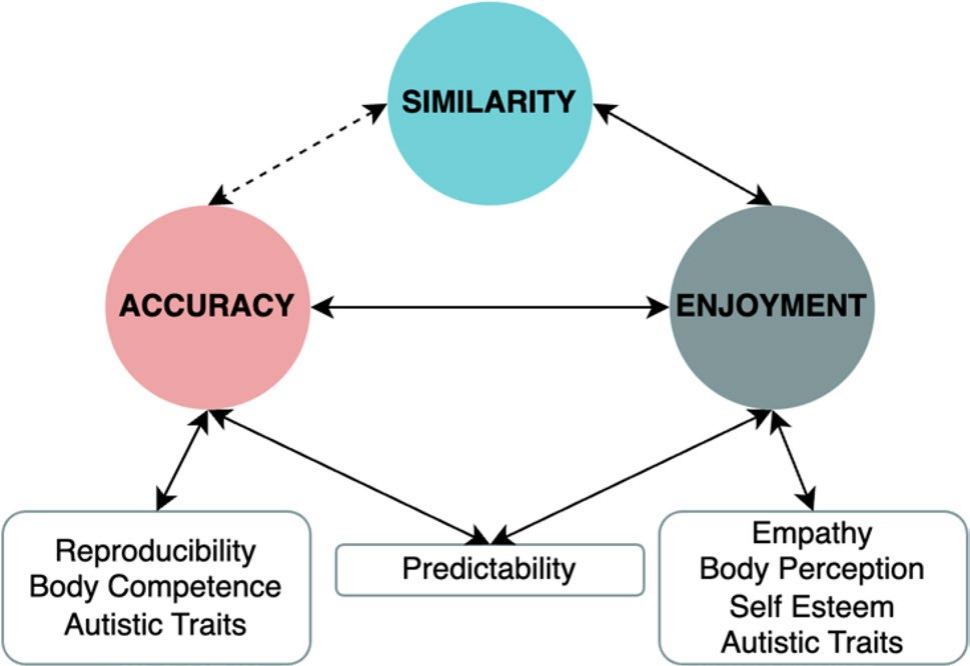
Schematic of observed relationships between variables. Similarity is to be interpreted as both a continuous variable (solid line to enjoyment) and a categorical variable (dotted line to accuracy), as per our models.

We set out to achieve two aims through this study. First, we sought to replicate previous findings that people are poor judges of the degree of motor synchrony in actions they observe, but that they derive more enjoyment from watching others’ actions when motor synchrony is present^23^. We successfully replicated these findings in two separate experiments. Second, we assessed the extent to which observers’ abilities to estimate the degree of synchrony and their enjoyment of synchronous movements were influenced by individual differences relating to embodiment, psychosocial traits, and social competencies. Here, we found that accuracy was closely linked to embodiment (i.e., reproducibility, predictability, and body competence). For enjoyment, we found that these ratings were predicted by empathy, while very low enjoyment tracked with higher body perception scores, as well as lower self-esteem and autistic trait scores (Fig. 6).

By analyzing two independent datasets, first separately as preregistered and subsequently together, we report the replicability of our findings candidly, laying the groundwork for further research. We believe the differences between the samples collected in Experiments 1 and 2 can be attributed to sampling error. We observed that results from analyses of both datasets aggregated are more similar to those of Experiment 2 than 1, which is logical in the context of the narrower distributions observed for all interindividual traits in Experiment 2 than 1 (Fig. 1A). As in the summary above, we will focus on the findings from our analyses of the aggregated data (N = 322), as this should be the most robust approach.

### Accuracy and enjoyment reduced for low-synchrony movement sequences

Across both experiments, observers underestimated the degree of synchrony in the dyadic mirror-game videos. This finding is aligned with existing evidence that observers struggle to accurately estimate the level of synchrony in multi-person movements^23^. We propose that this may stem from a potential propensity among observers not to weight shared stillness (e.g., of torso, shoulder joints, or head) strongly in their judgements of synchrony. Rather, observers might weight the extremities more strongly, as they offer richer and more salient cues. This aligns with eye-tracking studies suggesting that observers focus on extremities when seeking information about movement direction^79–81^, and switch between moving targets when tracking the identity of multiple moving items^82,83^.

Additional evidence that observers relied more on moving extremities than stable central body parts comes from our observation that observers’ underestimations were greater in magnitude for videos showing low, relative to high, amounts of synchrony (Fig. 1B). In the low synchrony videos, only one stick figure actively moved their extremities while the other was relatively still, giving participants fewer moving extremities with which to estimate the level of synchrony. Together, these corroborate the idea that the central still body parts are not strongly weighted in observers’ assessments of synchrony.

Other possibilities include that participants assign more weight to differences rather than similarities in movements or that the interplay between synchrony and enjoyment may influence accuracy when judging the degree of synchrony (Fig. 6). Observers’ accuracy when estimating synchrony and their ratings of enjoyment were positively correlated, particularly in the presence of a high degree of synchrony (Fig. 2). Vicary et al.^23^ and McEllin et al.^24^ each demonstrated that measured movement similarity predicts observers’ ratings of enjoyment. Vicary et al. provide additional evidence that observers’ accuracy correlates with the experience of strong affective responses (both positive and negative) to observed movement. According to our findings, high levels of synchrony are likely to induce greater positive affective responses, which may in turn increase observers’ sensitivity to synchrony. One limitation of the present study is that we did not assess accuracy as predicted by measured similarity (a continuous variable) but rather as predicted by high and low synchrony as categorical variables. This approach was not preregistered and was selected when visual inspection of the raw accuracy data (Fig. 1B) revealed the distribution to be bimodally conditioned by the type of mirroring task performed by the stick figures, rather than normally distributed (further details in Methods).

### Differences in accuracy may be driven by embodiment

Observers’ accuracy when estimating levels of synchrony was differentially associated with observers’ ratings of how well they could reproduce the movements and the measured predictability of movements for each level of synchrony. Greater reproducibility and predictability were associated with improved accuracy for movement sequences showing high synchrony and reduced accuracy for sequences showing low synchrony (Fig. 2). These measures offer complementary insights into the relationship between observers’ physical abilities and observed movement (what we refer to here as embodiment). Ratings of reproducibility shed light on the strength of an observer’s belief that they can physically reproduce an observed movement with their own body^40^. Predictability, on the other hand, offers an objective measure of the feasibility of a sequence of movements being represented within one’s body^25,39^. Our theoretical basis for our hypotheses was that greater embodiment is likely to support more accurate detection of synchrony^54^, and we did not anticipate this relationship to differ between low and high synchrony. One possible explanation for this unexpected difference is that observers’ focus on extremities may dampen the benefits that they could otherwise glean from greater embodiment of a movement sequence. When observers are attending to extremities belonging to both stick figures (high synchrony), observers’ embodiment helps represent both figures’ movements simultaneously, whereas when the extremities of one stick figure are less salient due to their stillness (low synchrony), observers may represent the more mobile figure more faithfully, potentially amplifying the differences in perceived motor synchrony.

Specifically for low-synchrony sequences, observers with greater body competence (i.e., belief in their bodily ability to accomplish desired movements competently) were more accurate in their estimations (Fig. 3). It is plausible that individuals with greater body competence scores have acquired some physical expertise, thereby honing their motor and motor-observation skills, which may reduce their reliance on extremities and enhance their attention to more subtle movements of the torso and head^58,84^. Specifically for high-synchrony sequences, we observed increasing underestimation with a greater number of autistic traits. An enduring debate has been held regarding a possible link between autism and reduced sensitivity to biological motion^73,75^, and this particular finding may be added to the mounting evidence.

In practical terms, people whose hobbies or livelihoods are focused on teaching or evaluating body movements (i.e., sports coaches, instructors, or adjudicators of performing arts) will require the ability to assess synchronicity and will likely draw upon their embodied expertise to do so. We base this proposition on existing work demonstrating that expert musicians are better able to perceive and maintain synchrony than non-experts, likely as a result of their embodiment of multisensory (auditory and visual) rhythmic knowledge^50–54^. Returning to perception of movement synchrony, we propose that lay observers, for whom this “skill” might not seem so important, may still benefit from the positive association between accuracy and enjoyment for highly synchronous movements. Perhaps most importantly, embodiment is cumulative^39,43^, meaning that enjoyment and accuracy could improve with repeated exposure to multi-body movements through embodied activities (e.g., yoga or dancing at a festival) or visual presentations thereof (e.g., dance trends on social media, documentaries about multi-athlete sports, or performing arts).

### Enjoyment shaped by movement predictability and individual differences

Given our prior (and preregistered) expectation that measured synchrony and enjoyment should correlate, we accounted for movement similarity when we modelled the influence of other parameters on enjoyment. Beyond the expected relationship between movement similarity and enjoyment, we found evidence for associations between enjoyment and movement predictability, as well as some individual traits. First, observers’ ratings of enjoyment were positively correlated with the measured predictability of movements (Fig. 5). This finding is consistent with previous work employing mirror-game movements to explore the aesthetic experience of observing synchronous movements^24^, where participants rated dots representing a hand-based mirror-game. Here, we have demonstrated that this relationship between the predictability of improvised movements and enjoyment holds for more complex visual stimuli, such as bodies showing biological motion. This relationship may, however, be specific to interactive movements. This would be consistent with Orlandi et al.’s^41^ findings that observers preferred less predictable movements performed by a single dancer.

We further observed that observers’ empathy scores predicted their enjoyment of movement sequences (Fig. 5); greater empathy was associated with greater enjoyment (differences in measured synchrony accounted for). It has been demonstrated that observers can make use of the degree of synchrony in shared movements to extract information about cohesion and social closeness^24,85–87^. Further, empathy has previously been associated with aesthetic appreciation of visual and performing art^88–90^. Considering these findings together, it seems likely that observers with greater empathy draw stronger connections between dyadic movement and the positive affect and social closeness that tend to emerge from engaging in synchronous movements.

Upon visually inspecting the enjoyment ratings, we were surprised by the relative prevalence of very low ratings (Fig. 1C), and consequently opted to model enjoyment in such a way that we could assess relationships between movement characteristics, ratings, interindividual traits, and the likelihood of very low enjoyment. Our analyses revealed that elevated body perception scores (a measure of awareness of bodily signals) predicted an increased likelihood of very low enjoyment. This relationship was observed across both experiments and for all data aggregated, suggesting that the relationship is quite robust. Our interpretation is that heightened attention to one’s bodily functions may reduce one’s bandwidth for social interaction or be symptomatic of disorders that reduce one’s bandwidth for social interaction, such as depression and schizophrenia^59–61^. It is plausible that greater awareness of one’s bodily signals inhibits attention to socially relevant kinematic information, thereby potentially weakening the representation and enjoyment of movements performed by other social agents.

We acknowledge that this interpretation contradicts recent findings linking greater attention to bodily signals and to stronger empathetic tendencies^91,92^. However, it must also be noted that this recent work does not incorporate any elements of social interaction with which to assess whether the observed relationship is relevant in social scenarios. It would be valuable for future work to explore this relationship explicitly. Considering this, a potential alternative explanation for our findings could be that individuals who attend more closely to their bodily sensations may also perceive mirror-game movements as less interesting or enjoyable in comparison to complex dance movements or sport performances. From this perspective, such individuals may require more dramatic and arousal-inducing displays of movement to experience enjoyment^93^.

Returning to the idea that individuals who pay more attention to their bodies may have reduced capacity to extract bodily information from social interactions, we draw on studies of neural and behavioural synchrony, or coupling, between and within individuals. Whereas greater interpersonal neural coupling results in reduced intrapersonal neural coupling^94,95^, it may be the case that stronger intrapersonal neural coupling may reduce an individual’s openness to or propensity for interpersonal coupling on neural and behavioral levels^18^. Braiding these avenues of speculation together, we suggest that observers whose sensory attention is directed inward (i.e., intrapersonal neural coupling) may be less inclined to notice behavioral interpersonal coupling from a third-person perspective. This reasoning fits with Shamay-Tsoory et al.’s^18^ model of alignment, in which bodily, cognitive, and emotional coupling are maintained via ‘gap-detection’. In other words, increased attention to one’s bodily signals could consume attentional resources otherwise allocated to detecting gaps in interpersonal coupling. Further investigations of the interplay between ‘gap-detection’, behavioral coupling, and neural coupling are needed to confirm these speculations.

Additionally, we observed that higher scores for extraversion, self-esteem, body competence, and empathy were each associated with a reduced likelihood of very low enjoyment. Of these, self-esteem and autistic traits showed the strongest relationship with very low enjoyment (Fig. 4). We suspect that observers with higher levels of traits related to outward orientation (i.e., extraversion, self-esteem, body competence, and empathy) may plausibly form stronger representations of the observed social interactions. Perhaps, this may result from more practice or more comfort with interpersonal coupling^25,65^. In everyday life, this may mean that strength of an observer’s outward orientation may predict their aesthetic preferences when observing people moving together (e.g., greater enjoyment of coordinated/synchronized movements during a dance or orchestral performance than movements made during a soccer game, which might be considered enjoyable for other reasons).

## Conclusion

We examined observers’ abilities to estimate the degree of motor synchrony in short dyadic mirror-game sequences and their enjoyment thereof, considering characteristics of the movements and individual trait differences. Our findings demonstrate that accuracy is contingent on the observer’s embodiment, while enjoyment of the aesthetics of synchronized actions is shaped by interindividual differences including empathy, body perception, self-esteem, and autistic traits. With this foundation, future research can delve into the mechanisms that drive how we, as diverse and unique observers, perceive and respond to motor synchrony in everyday life.

## Method

### Participants

In our preregistrations for each experiment, we stated that we would collect 160 useable datasets. To do so, we replaced participants who failed to complete the entire experiment or failed 2–3 of 3 attention check questions. In our initial round, 206 participants were recruited from Macquarie University’s undergraduate psychology-student pool to obtain useable data from 161 participants (108 female; 49 male; 2 other; 2 prefer not to say; mean age = 24.28 ± 9.09). For Experiment 2, 201 new participants were recruited from the same student pool to obtain useable data from another 161 participants (124 female; 36 male; 1 other; mean age = 21.75 ± 9.43). As offline data quality checks lagged a day behind online data collection, both samples contain one participant more than we preregistered. Participants were not permitted to complete both experiments, meaning that each sample contains different participants.

Ethical approval for this study was obtained from the Macquarie University Human Research Ethics Committee (Ref: 520231198949711). All participants provided written informed consent and this research was undertaken in accordance with the Declaration of Helsinki. Participants received course credit or a cash honorarium (AUD $20) for their involvement.

### Individual trait measures

Extraversion was measured using the International Personality Item Pool (IPIP) representations of the extraversion subscale of the Goldberg^96^ Big Five markers. We measured self-esteem using the Rosenberg^97^ self-esteem scale. Body awareness was recorded using the Body Perception Questionnaire (BPQ)^57^. Body competence was assessed using the Body Competence subscale of the Body Consciousness Questionnaire (BCQ)^56^. Empathy was assessed using the total score of the Interpersonal Reactivity Index (IRI)^98^ and autistic traits were measured using the Comprehensive Autistic Trait Inventory (CATI)^99^. All measures used 5-point Likert scales, except the self-esteem measure, which used a 4-point Likert scale. Refer to Table 1 for example items and associated Likert-scale labels.

**Table 1:** Interindividual measures recorded in online questionnaire.

Scale	Scale endpoints	Example items
Extraversion^96^	1 = very inaccurate 5 = very accurate	‘I feel comfortable around people’ ‘I don’t talk a lot’
Self-esteem^97^	0 = strongly disagree 3 = strongly agree	‘I know my strengths’ ‘I am less capable than most people’
Body awareness^57^	1 = never 5 = always	‘Swallowing frequently’ ‘My mouth being dry’
Body competence^56^	0 = extremely uncharacteristic 4 = extremely characteristic	‘For my size, I'm pretty strong’ ‘I'm capable of moving quickly’
Empathy^98^	0 = does not describe me well 4 = describes me very well	‘I am often quite touched by things that I see happen’ ‘…I don't feel very sorry for other people…having problems’
Autistic traits^99^	1 = definitely disagree 5 = definitely agree	‘I like to stick to certain routines for every-day tasks’ ‘I generally enjoy social events’

### Video stimuli 

**Figure 7:**
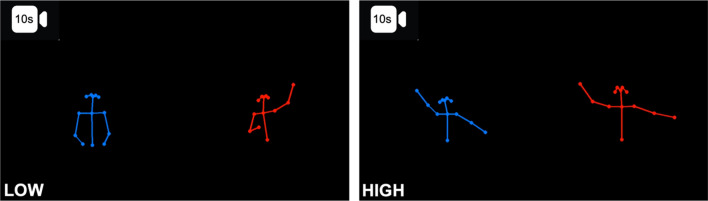
Stills from 10-s stimulus videos depicting low (left) and high (right) synchrony conditions. Low synchrony videos show one person moving their arms while the other observes. High synchrony videos show the dyad playing the mirror game, where one person moves their arms and the other matches the movements as closely as possible.

Participants viewed 10-s videos showing two stick figures, one red and one blue, moving their arms in varying degrees of synchrony. By using stick figures, as opposed to videos of real people, we could mitigate biases resulting from differences in appearance (i.e., gender, skin tone, facial expressions) which may influence accuracy^22^. These stimulus videos (n = 198) were generated from longer videos of real human dyads playing the mirror game with a partner (high synchrony; 85% of videos) or observing their partner’s movements (low synchrony; 15% of videos) in a previous experiment^16^. Our intention was to use the level of objectively measured movement synchrony as one continuous variable. However, upon inspecting the data, we observed a bimodal distribution matching the underlying task in the video (mirroring vs. observing) and determined it would be necessary to account for participants’ sensitivity to this bimodal distribution in our analysis. To address this, we labelled mirroring videos “high synchrony” and movement observation videos “low synchrony”, turning the level of synchrony into a categorical variable. To generate the videos, the positions of dyad’s body parts in the original videos were estimated, per frame, using OpenPose software^100^. These coordinates were wrangled into a time series using code adapted from de Jonge-Hoekstra (https://osf.io/6s73d/) and then smoothed using a Savitzky-Golay filter (window length = 13 frames; polynomial order = 2) implemented with the signal R package (version 0.7–7)^101^. Next, for each frame, the time series per body part was mapped onto a black background using dots joined by lines, creating stick figures using Pillow python package^102^. The frames were appended to each other to create videos using python package OpenCV (version 4.5.5.62)^103^. These were divided into 10-s segments using ffmpeg^104^. The 10-s videos were screened for motion-capture artefacts by R.M. and a junior lab member, yielding a set of 198 videos (Fig. 7).

The 198 videos were drawn from 22 unique dyads in our previous experiment^16^, where each participant played the mirror game with an experimental confederate. Among the 22 dyads, the mirror game was led by 16 unique individuals, i.e., the participant was the leader of the mirror game in 14 dyads and the two experimental confederates were the leaders of the mirror game in the remaining dyads. We included a range of unique leaders to ensure a variety of movement patterns in the videos, thereby enhancing the generalizability of the stimuli. To control for potential response biases related to correspondences between the color or position of the leading stick figures, we generated the videos so that the leader and follower were each red in 50% of the videos and blue in the other 50%, and across these, positioned on the left or the right in 50% of videos. In other words, leaders, followers, and dyads cannot be linked to a spatial or color-based pattern.

To lighten the download demands of the online experiment, we divided the set of videos into two equal groups of 98 unique videos, with additional 2 videos being the same in both groups, to create subsets (A and B) of 100 videos. We carefully assigned 50% of the videos per leader to each subset, so that all leaders appear in both subsets. We paid special attention to maintain the spatial and color-based counterbalancing described above.

### Objective kinematic measures

We extracted objective measures of movement similarity and predictability for each 10-s video from the time series of coordinates extracted from OpenPose. We objectively quantified movement similarity using R code adapted from Broadwell & Tangherlini^105^. Broadwell & Tangherlini’s approach captures the similarity of whole body, or in our case, upper body poses of two or more people per video frame, maximizing the richness of the spatial and temporal information encoded in the similarity score. The first step was to estimate the Euclidian distance between pairs of a figure’s neck, shoulders, elbows, and hands per frame [i.e., calculate distances between each combination of these body parts (n = 42) for red figure, then do the same for the blue figure]. These distances were then stored in a separate ‘pose matrix’ per figure. The distances stored in the matrices are body-centric, that is, normalized within the matrix, and as such, not influenced by differences in height or position relative to the camera. To establish similarity per frame, the matrices were compared (i.e., blue figure vs. red figure), returning a value between 0 = no similarity and 1 = identical per frame. We extracted the mean similarity across the 600 frames for each video. For additional details, see Broadwell & Tangherlini’s^105^ implementation of this measure on groups of K-Pop dancers.

To obtain a measure of movement predictability, we calculated the sample entropy^106^ using the R package pracma (version 2.4.2)^107^. Sample entropy was calculated for the timeseries of the x- and y- coordinates of each the right and left wrist of each member of a dyad across each 10-s video. Eight entropy values were obtained (2 coordinates*2 hands*2 people) and averaged per video. Entropy values, are henceforth referred to as ‘predictability’, with values closer to 0 indicating increased ‘purity’ of the signal, or greater predictability^41,106^.

### Subjective aesthetic measures

After viewing the videos, participants provided subjective ratings on three scales. We assessed participants’ perceptions of similarity within a dyad’s upper body movements using the question ‘How IN SYNCH were the people in this video?’. Participants’ aesthetic appreciation of the movements was probed via the question ‘How much did you ENJOY watching the movements in this video?’. Finally, participants’ perceived ability to reproduce the movements (a measure of perceived embodiment) was acquired through the question ‘How identically could you REPRODUCE these movements with your body?’. Participants responded using their computer mouse on a sliding scale ranging from ‘not at all’ to ‘completely’. These labels corresponded to numeric values from 0 through 100, which were not visible to the participants.

### Procedure

Participants completed the experiment online, requiring approximately 40 min. First, participants completed a questionnaire recording demographic information and individual measures (as detailed in Table 1), administered via Redcap®. Upon finishing, participants were automatically redirected to a video-rating task presented via Pavlovia. For both experiments, the video-rating task was launched with stimuli from set A. After approximately 80 useable datasets were obtained, the stimuli were switched to set B for the remainder of the data collection period.

The video-rating task consisted of 153 trials (150 real trials and 3 attention checks). Per participant, 50 videos were randomly assigned from the stimulus set of 100 videos (A or B). To enable participants to respond to each of the 3 subjective rating questions for each of the 50 videos, the 50 videos were each repeated 3 times. In other words, participants viewed 150 videos in a randomized order and answered one rating question per video, where the order of questions was also randomized. The presentation of three additional videos was followed by an attention-checking question ‘How much did these stick figures resemble automobiles?’. The correct response was ‘not at all’, corresponding to 0 on the sliding scale, and ratings < 0–25/100 were accepted as correct. Data were included if participants responded correctly to at least 2/3 attention questions.

### Data analysis

The influence of kinematic measures and individual traits on how accurately participants estimated synchrony was assessed using R (version 4.3.1)^108^ in the RStudio IDE (version 2022.07.2)^109^. We fit Bayesian multilevel models using R package brms (version 2.20.1)^110^. Z-scores were taken for all predictor variables, to homogenize the scale of each predictor, facilitating the interpretation and comparison of parameter estimates. In our analyses of accuracy, we implemented distributional models, which were useful in that we could estimate each parameter (kinematic and trait measures) for high and low synchrony separately, within one model. To assess the extent to which kinematic measures and individual traits were related to participants’ enjoyment, we employed zero-inflated gaussian models. This allowed for very low enjoyment (ratings 0–10/100) and enjoyment, as a continuous construct (ratings 11–100/100) to be assessed independently from each other within the same model. We report our full models, comparison to simpler models, and visualization of all model parameters in Sect.  2 of Supplementary Material.

Our preregistered approach to analyzing the data from each experiment was to fit models for each experiment separately, should > 100 useable data sets be collected for Experiment 2, or in the case that < 100 useable data sets could be collected, to aggregate the data with that from Experiment 1. Here, we first report the results for each experiment, as per our preregistration. Subsequently, we present the results from exploratory analyses in which we fit the same models to the aggregated data, to illustrate which findings are likely to be most robust to sampling error.

## Supplementary Information

Supplementary Information.

## Data Availability

The datasets collected and analyzed for this study can be found our repository entitled “Perceptions of synchronous movement” on OSF: https://osf.io/ugczs/.
