# Author Correction: Evaluations of dyadic synchrony: observers’ traits influence estimation and enjoyment of synchrony in mirror-game movements

**DOI:** 10.1038/s41598-025-98358-5

**Published:** 2025-04-23

**Authors:** Ryssa Moffat, Emily S. Cross

**Affiliations:** 1 Professorship for Social Brain Sciences, ETH Zurich, Zurich, Switzerland; 2 School of Psychological Sciences, Macquarie University, Sydney, NSW Australia; 3 MARCS Institute, Western Sydney University, Sydney, NSW Australia

Correction to: *Scientific Reports* 10.1038/s41598-024-53191-0, published online 05 February 2024

The original version of this Article contained an error in the interpretation of the relationship between austistic traits and estimation of synchrony. The text of published article incorrectly indicated that greater autistic traits are associated with improved accuracy. The corrected text indicates that more numerous autistic traits are associated with greater underestimation (i.e., more autistic traits are associated with decreased accuracy).

As the result, in the Abstract,

“Accuracy for low synchrony improved with increasing body competence, while accuracy for high synchrony improved with increasing autistic traits.”

now reads:

“Accuracy for low synchrony improved with increasing body competence, while underestimation for high synchrony rose with increasing autistic traits.”

In the Results section, under the subheading ‘Greater body competence improves accuracy for low-synchrony movements’,

“Further, for low synchrony, Experiment 2 showed trending associations between improved accuracy and greater self-esteem (96% of HPD below 0) and more autism traits (94% of HPD below 0; Supplementary Table 4). The same model, fit to the aggregated data from Experiments 1 and 2, indicated the same negative relationship between body competence and accuracy for low synchrony, as well as positive relationship between autistic traits and accuracy for high synchrony (Fig. 3).”

now reads:

“Further, for low synchrony, Experiment 2 showed trending associations between improved accuracy and greater self-esteem (96% of HPD below 0) as well as reduced accuracy and more autism traits (94% of HPD below 0; Supplementary Table 4). The same model, fit to the aggregated data from Experiments 1 and 2, indicated the same negative relationship between body competence and accuracy for low synchrony, as well as positive relationship between autistic traits and greater underestimation for high synchrony (Fig. 3).”

Finally, in the Discussion, under the subheading ‘Differences in accuracy may be driven by embodiment’,

“Specifically for high-synchrony sequences, we observed increasing accuracy with a greater number of autistic traits. An enduring debate has been held regarding a possible link between autism and reduced sensitivity to biological motion^73,75^, and this particular finding may be added to the counter-evidence.”

now reads:

“Specifically for high-synchrony sequences, we observed increasing underestimation with a greater number of autistic traits. An enduring debate has been held regarding a possible link between autism and reduced sensitivity to biological motion^73,75^, and this particular finding may be added to the mounting evidence.”

The original Article has been corrected.

