# Supplementary material for: Evaluations of dyadic synchrony: observers’ traits influence estimation and enjoyment of synchrony in mirror-game movements
Source: Sci Rep. 2024 Feb 5;14:2904. doi: 10.1038/s41598-024-53191-0 (PMC10844651; doi:10.1038/s41598-024-53191-0)

SUPPLEMENTARY MATERIALS
Evaluations of dyadic synchrony: Observers' traits influence estimation and enjoyment of synchrony in mirror-game movements

Section 1 – Descriptive statistics and parameter estimates

Supplementary Table 1: Descriptive statistics for interindividual measures. See Figure 1A in main manuscript for depiction of distributions. SD = standard deviation.

	Experiment 1		Experiment 2	
**Measure**	**Mean**	**SD**	**Mean**	**SD**
**Extraversion**	30.61	7.90	30.89	6.80
**Self-Esteem**	18.39	5.35	17.80	3.79
**Body Perception**	66.57	19.07	65.39	17.38
**Body Competence**	9.40	3.39	9.11	2.76
**Empathy**	69.65	13.78	70.32	11.88
**Autistic Traits**	113.63	26.27	109.43	22.10

Supplementary Table 2: Parameter estimates for Accuracy (measured movement similarity - estimated movement similarity) overall, as well as for low and high synchrony. HPD = 95% highest posterior density region.

	Experiment 1		Experiment 2	
**Synchrony**	**Estimate**	**HPD**	**Estimate**	**HPD**
**Mean**	28.30	26.90, 29.70	26.90	25.40, 28.40
**Low**	43.00	41.30, 44.70	42.60	40.82, 44.40
**High**	13.60	12.30, 15.00	11.30	9.84, 12.70

Supplementary Table 3: Relationship between accuracy and measured predictability and similarity of movements, per degree of synchrony. HPD = 95% highest posterior density region.

		Experiment 1		Experiment 2		Experiment 1+2	
**Measure**	**Synchrony**	**Estimate**	**HPD**	**Estimate**	**HPD**	**Estimate**	**HPD**
**Predictability**	Low	-1.17	-2.37, -0.02	-2.61	-3.78, -1.41	-1.93	-2.77, -1.10
	High	1.05	0.60, 1.50	0.32	-0.12, 0.76	0.69	0.38, 1.02
**Enjoyment**	Low	1.30	0.20, 2.41	-0.23	-1.36, 0.92	-0.58	-0.23, 1.37
	High	-2.31	-2.96, -1.69	-2.58	-3.22, 1.97	-2.46	-2.90, -2.00
**Reproducibility**	Low	0.43	-0.66, 1.49	2.22	1.01, 3.47	1.38	0.53, 2.18
	High	-1.37	-2.05, -0.70	-2.01	-2.69, -1.28	-1.65	-2.16, -1.17

Supplementary Table 4: Relationship between accuracy and interindividual measures, per degree of synchrony. HPD = 95% highest posterior density region.

		Experiment 1		Experiment 2		Experiment 1+2	
**Measure**	**Synchrony**	**Estimate**	**HPD**	**Estimate**	**HPD**	**Estimate**	**HPD**
**Extraversion**	Low	1.02	-0.61, 2.69	-0.17	-2.03, 1.74	-0.87	-2.34, 0.56
	High	0.86	-0.51, 2.16	0.74	-0.77, 2.33	0.55	-0.64, 1.77
**Self-esteem**	Low	-0.91	-2.70, 0.86	-2.38	-4.46, 0.21	-0.91	-2.51, 0.67
	High	0.38	-1.08, 1.79	-0.81	-2.56, 0.96	-0.12	-1.44, 1.17
**Body Perception**	Low	-1.41	-3.00, 0.79	-1.01	-2.96, 0.89	-0.90	-2.31, 0.57
	High	-0.42	-1.91, 1.17	-0.16	-1.41, 1.75	0.10	-1.11, 1.29
**Body Competence**	Low	-1.77	-3.48, -0.12	-1.46	-3.28, 0.47	-1.49	-2.85, -0.07
	High	0.33	-1.07, 1.68	0.28	-1.23, 1.85	0.34	-0.82, 1.50
**Empathy**	Low	0.57	-1.29, 2.44	0.69	-1.17, 2.53	0.56	-0.77, 1.91
	High	0.79	-0.77, 2.30	-0.93	-2.41, 0.58	-0.69	-1.81, 0.42
**Autistic Traits**	Low	1.00	-1.08, 2.96	-2.46	-4.63, 0.32	-0.87	-2.49, 0.68
	High	0.79	-0.85, 2.47	1.12	-0.69, 2.87	1.37	0.07, 2.72

Supplementary Table 5: Relationship between very low enjoyment and interindividual measures. HPD = 95% highest posterior density region. For Experiment 1+2: 10 % of HPD for body perception is above 0.

	Very low enjoyment (0-10/100)					
	Experiment 1		Experiment 2		Experiment 1+2	
**Measure**	**Estimate**	**HPD**	**Estimate**	**HPD**	**Estimate**	**HPD**
**Extraversion**	-0.20	-0.26, -0.14	-0.11	-0.18, -0.04	-0.07	-0.12, -0.02
**Self-Esteem**	0.15	0.08, 0.22	-0.34	-0.43, -0.27	-0.31	-0.36, -0.25
**Body Percep.**	0.31	0.23, 0.38	-0.08	-0.16, -0.01	0.10	0.05, 0.15
**Body Compet.**	0.11	0.05, 0.17	-0.32	-0.38, -0.25	-0.04	-0.09, 0.01
**Empathy**	-0.17	-0.24, -0.10	0.06	-0.01, 0.13	-0.08	-0.12, -0.03
**Autistic Traits**	-0.24	-0.33, -0.17	-0.34	-0.42, -0.26	-0.28	-0.33, -0.22
**Similarity**	-0.04	-0.10, 0.22	-0.08	-0.15, -0.02	-0.05	-0.10, -0.01
**Predictability**	0.05	-0.01, 0.11	0.00	-0.07, 0.06	0.02	-0.02, 0.06

Supplementary Table 6: Relationship between enjoyment per se and interindividual measures. HPD = 95% highest posterior density region. Experiment 1+2: for enjoyment per se, 5% of empathy HPD is below 0. Body Compet. = Body competence; Body Percep. = Body perception.

	Enjoyment per se (11-100/100)					
	Experiment 1		Experiment 2		Experiment 1+2	
**Measure**	**Estimate**	**HPD**	**Estimate**	**HPD**	**Estimate**	**HPD**
**Extraversion**	2.23	0.36, 4.00	0.02	-1.76, 2.02	0.48	-1.07, 2.01
**Self-Esteem**	0.38	-1.62, 2.25	2.87	0.68, 4.92	2.84	1.11, 4.59
**Body Percep.**	-1.07	-3.19, 1.09	2.65	0.70, 4.64	1.03	-0.63, 2.62
**Body Compet.**	-0.81	-2.57, 1.06	2.81	0.94, 4.58	0.31	-1.20, 1.81
**Empathy**	1.70	-0.36, 3.68	2.03	0.24, 3.85	1.29	-0.15, 2.79
**Autistic Traits**	-1.89	-4.10, 0.31	-0.40	-2.56, 1.78	-0.80	-2.60, 0.89
**Similarity**	0.50	0.06, 0.93	1.22	0.80, 1.67	0.86	0.56, 1.16
**Predictability**	-0.76	-1.19, 0.33	-1.04	-1.46, -0.62	-0.90	-1.20, -0.60

Section 2 – Full models predicting accuracy and enjoyment of synchronous dyadic movements

Supplementary Figure A) Influence of enjoyment, reproducibility, and predictability on accuracy: Model comparison using LOO (leave-one-out cross-validation). In both experiments, H4.0 is the worst model. Error bars for all other models overlap greatly, suggesting that no model offers uniquely better predictive accuracy. elpd_loo = expected log pointwise predictive density, a way of ranking models by predictive accuracy.

`simDif` = objectively measured similarity – estimated similarity
`Moving` = categorical variable high or low synchrony
`enjoyment_z` = z-scored ratings of enjoyment
`reproducibility_z` = z-scored ratings of reproducibility
`M_entropy1_z` = z-scored and averaged entropy of right and left wrists

- *H4.0: simDif ~ 1 + Moving, sigma ~ Moving*
- *H4.1: simDif ~ 1 + Moving + (1|ID), sigma ~ Moving*
- *H4.2: simDif ~ 1 + Moving + M_entropy1_z + (1|ID), sigma ~ Moving*
- *H4.3: simDif ~ 1 + Moving + enjoyment_z + M_entropy1_z + (1|ID), sigma ~ Moving*
- *H4.4: simDif ~ 1 + Moving + enjoyment_z + reproducibility_z + M_entropy1_z + (1|ID), sigma ~ Moving*
- *H4.5: simDif ~ 1 + Moving * (enjoyment_z) + reproducibility_z + M_entropy1_z + (1|ID), sigma ~ Moving*
- *H4.6: simDif ~ 1 + Moving * (enjoyment_z + reproducibility_z) + M_entropy1_z + (1|ID), sigma ~ Moving*
- *H4.7: simDif ~ 1 + Moving * (enjoyment_z + reproducibility_z + M_entropy1_z) +(1|ID), sigma ~ Moving*
- *H4.8: simDif ~ 1 + Moving * (enjoyment_z*reproducibility_z + M_entropy1_z) + (1|ID), sigma ~ Moving*

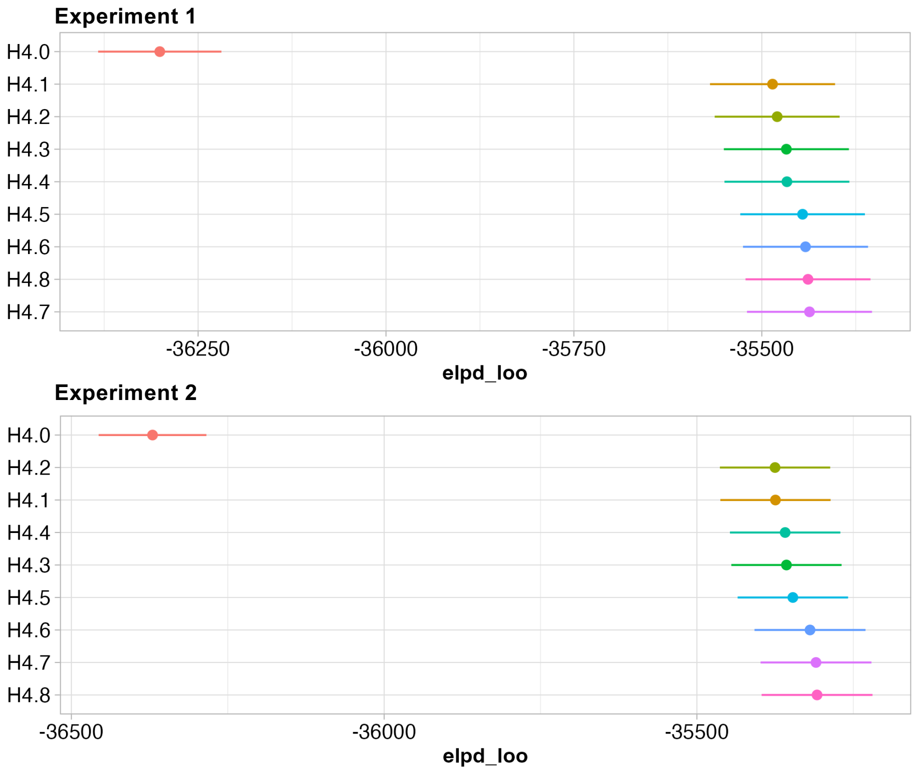

Supplementary Figure B) Influence of enjoyment, reproducibility, and predictability on accuracy: Parameter estimates from H4.8. Entropy is our measure of predictability, where greater positive values represent less predictability and smaller values represent a 'more pure', more predictable, signal.

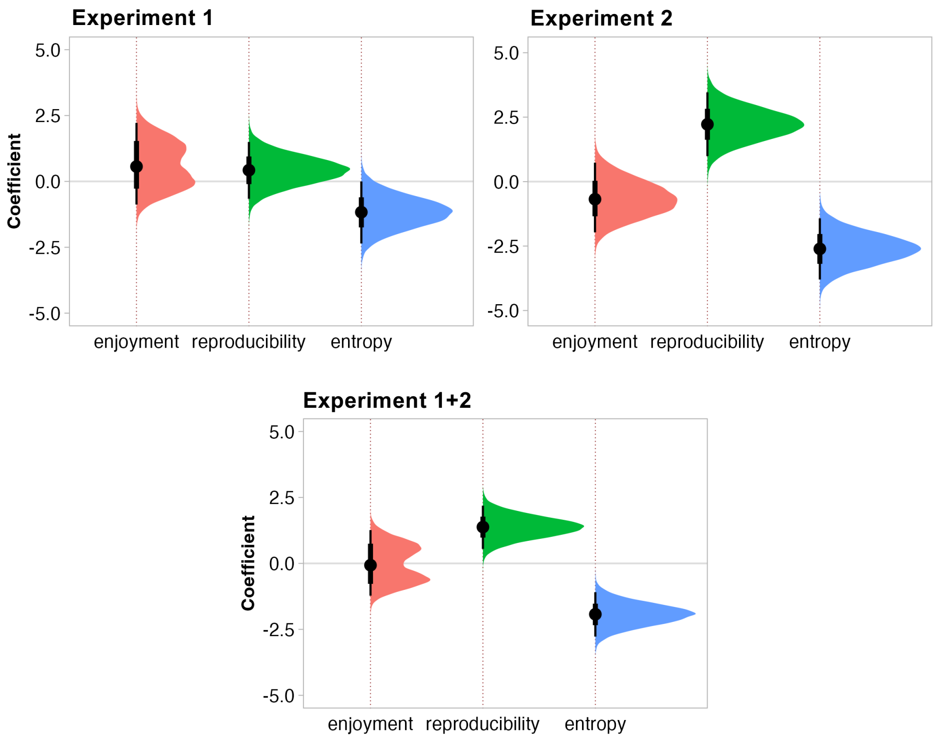

Supplementary Figure C) Influence of interindividual traits and predictability on accuracy: Model comparison using LOO (leave-one-out cross-validation). In both experiments, H3.0 is the worst model. Error bars for all other models overlap greatly, suggesting that no model offers uniquely better predictive accuracy.
elpd_loo = expected log pointwise predictive density, a way of ranking models by predictive accuracy.

`simDif` = objectively measured similarity – estimated similarity
`Moving` = categorical variable high or low synchrony
`extraversion_z` = z-scored extraversion score
`selfEsteem_z` = z-scored selfesteem score
`bpq_z` = z-scored body perception score
`bcq_z` = z-scored body competence score
`iri_z` = z-scored empathy score
`cati_z` = z-scored autistic trait score
`M_entropy1_z` = z-scored and averaged entropy of right and left wrists

- *H3.0: simDif ~ 1 + Moving, sigma ~ Moving*
- *H3.1: simDif ~ 1 + Moving + (1|ID), sigma ~ Moving*
- *H3.2: simDif ~ 1 + Moving + M_entropy1_z + (1|ID), sigma ~ Moving*
- *H3.3: simDif ~ 1 + Moving + extraversion_z + M_entropy1_z + (1|ID), sigma ~ Moving*
- *H3.4: simDif ~ 1 + Moving + extraversion_z + selfEsteem_z + M_entropy1_z + (1|ID), sigma ~ Moving*
- *H3.5: simDif ~ 1 + Moving + extraversion_z + selfEsteem_z + bpq_z + M_entropy1_z + (1|ID), sigma ~ Moving*
- *H3.6: simDif ~ 1 + Moving + extraversion_z + selfEsteem_z + bpq_z + bcq_z + M_entropy1_z + (1|ID), sigma ~ Moving*
- *H3.7: simDif ~ 1 + Moving + extraversion_z + selfEsteem_z + bpq_z + bcq_z + iri_z + M_entropy1_z + (1|ID), sigma ~ Moving*
- *H3.8: simDif ~ 1 + Moving + extraversion_z + selfEsteem_z + bpq_z + bcq_z + iri_z + cati_z + M_entropy1_z + (1|ID), sigma ~ Moving*
- *H3.9: simDif ~ 1 + Moving * (extraversion_z) + selfEsteem_z + bpq_z + bcq_z + iri_z + cati_z + M_entropy1_z + (1|ID), sigma ~ Moving*
- *H3.10: simDif ~ 1 + Moving * (extraversion_z + selfEsteem_z) + bpq_z + bcq_z + iri_z + cati_z + M_entropy1_z + (1|ID), sigma ~ Moving*
- *H3.11: simDif ~ 1 + Moving * (extraversion_z + selfEsteem_z + bpq_z) + bcq_z + iri_z + cati_z + M_entropy1_z + (1|ID), sigma ~ Moving*
- *H3.12: simDif ~ 1 + Moving * (extraversion_z + selfEsteem_z + bpq_z + bcq_z) + iri_z + cati_z + M_entropy1_z + (1|ID), sigma ~ Moving*
- *H3.13: simDif ~ 1 + Moving * (extraversion_z + selfEsteem_z + bpq_z + bcq_z + iri_z) + cati_z + M_entropy1_z + (1|ID), sigma ~ Moving*
- *H3.14: simDif ~ 1 + Moving * (extraversion_z + selfEsteem_z + bpq_z + bcq_z + iri_z + cati_z) + M_entropy1_z + (1|ID), sigma ~ Moving*
- *H3.15: simDif ~ 1 + Moving * (extraversion_z + selfEsteem_z + bpq_z + bcq_z + iri_z + cati_z + M_entropy1_z) + (1|ID), sigma ~ Moving*

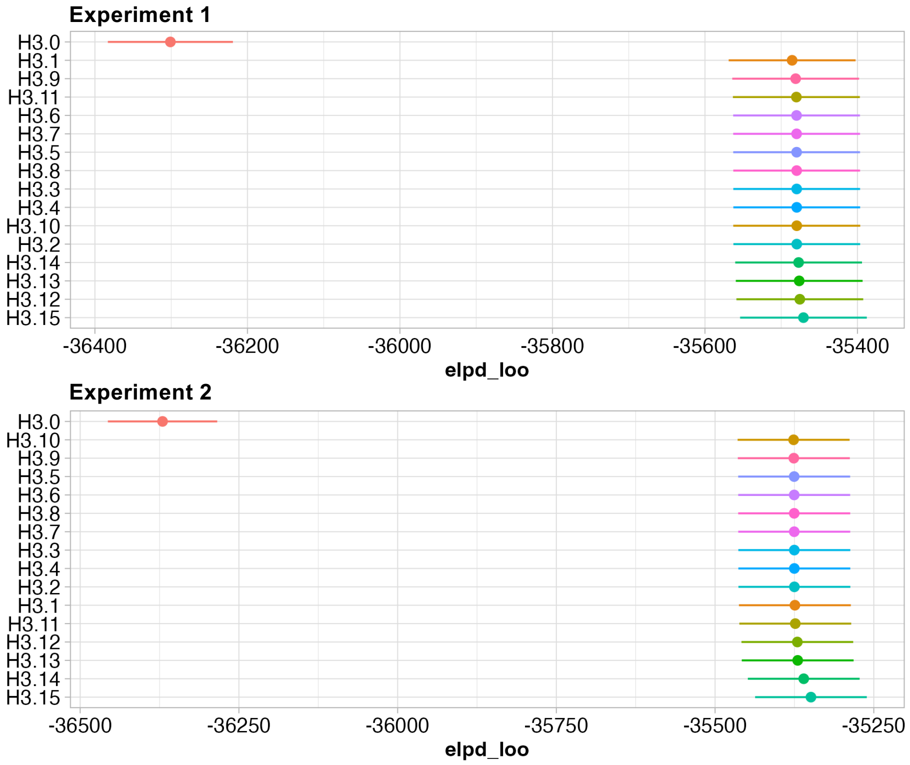
Experiment 1
H3.0
H3.1
H3.9
H3.11
H3.6
H3.7
H3.5
H3.8
H3.3
H3.4
H3.10
H3.2
H3.14
H3.13
H3.12
H3.15
-36400
-36200
-36000
-35800
-35600
-35400
elpd_loo
Experiment 2
H3.0
H3.10
H3.9
H3.5
H3.6
H3.8
H3.7
H3.3
H3.4
H3.2
H3.1
H3.11
H3.12
H3.13
H3.14
H3.15
-36500
-36250
-36000
-35750
-35500
-35250
elpd_loo

Supplementary Figure D) Influence of interindividual traits and predictability (i.e., entropy) on accuracy: Parameter estimates from H3.15. bpq = body perception; bcq = body competence; iri = empathy; cati = autistic traits.

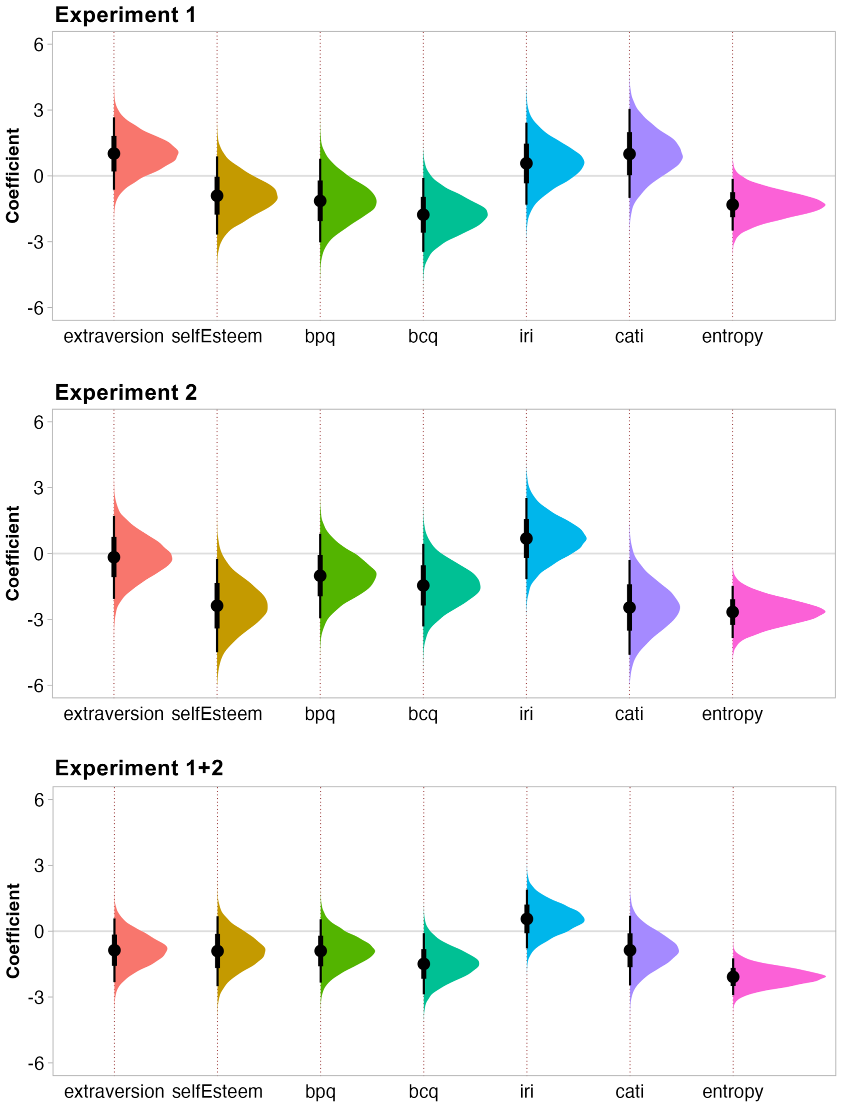

Supplementary Figure E) Influence of interindividual traits, movement similarity, and predictability on enjoyment. We used a hurdle-gaussian model to calculate the likelihood of very low enjoyment (ratings <11) for each trait, as well as the relationships between traits and ratings of enjoyment per se (ratings 11-100) in a single model. No model comparison using LOO (leave-one-out cross-validation) possible, as the *hurdle_gaussian* custom family from Andrew Heiss' tutorial (https://www.andrewheiss.com/blog/2022/05/09/hurdle-lognormal-gaussian-brms/) has yet to be integrated into the *brms* package. Our final model was:

```
Exp1.17: enjoyment_H ~ 1 + M_entropy1_z + M_similarity1_z + extraversion_z + selfEsteem_z + bpq_z + bcq_z + iri_z + cati_z + (1|ID), hu ~ 1 + M_entropy1_z + M_similarity1_z + extraversion_z + selfEsteem_z + bpq_z + bcq_z + iri_z + cati_z)
```

`Enjoyment_H` = enjoyment, where all ratings <11 out of 100 are replaced with 0
`M_entropy1_z` = z-scored and averaged entropy of right and left wrists
`M_similarity_z` = z-scored and averaged similarity of movements
`extraversion_z` = z-scored extraversion score
`selfEsteem_z` = z-scored selfesteem score
`bpq_z` = z-scored body perception score
`bcq_z` = z-scored body competence score
`iri_z` = z-scored empathy score
`cati_z` = z-scored autistic trait score
`hu` = the intercept representing the likelihood of very low enjoyment (ratings <11 out of 100)

Supplementary Figure F) Influence of interindividual traits, enjoyment, reproducibility, and predictability (entropy) on enjoyment: Hu (very low enjoyment, i.e., ratings of enjoyment that were <11 out of 100) parameter estimates from Exp1.17.

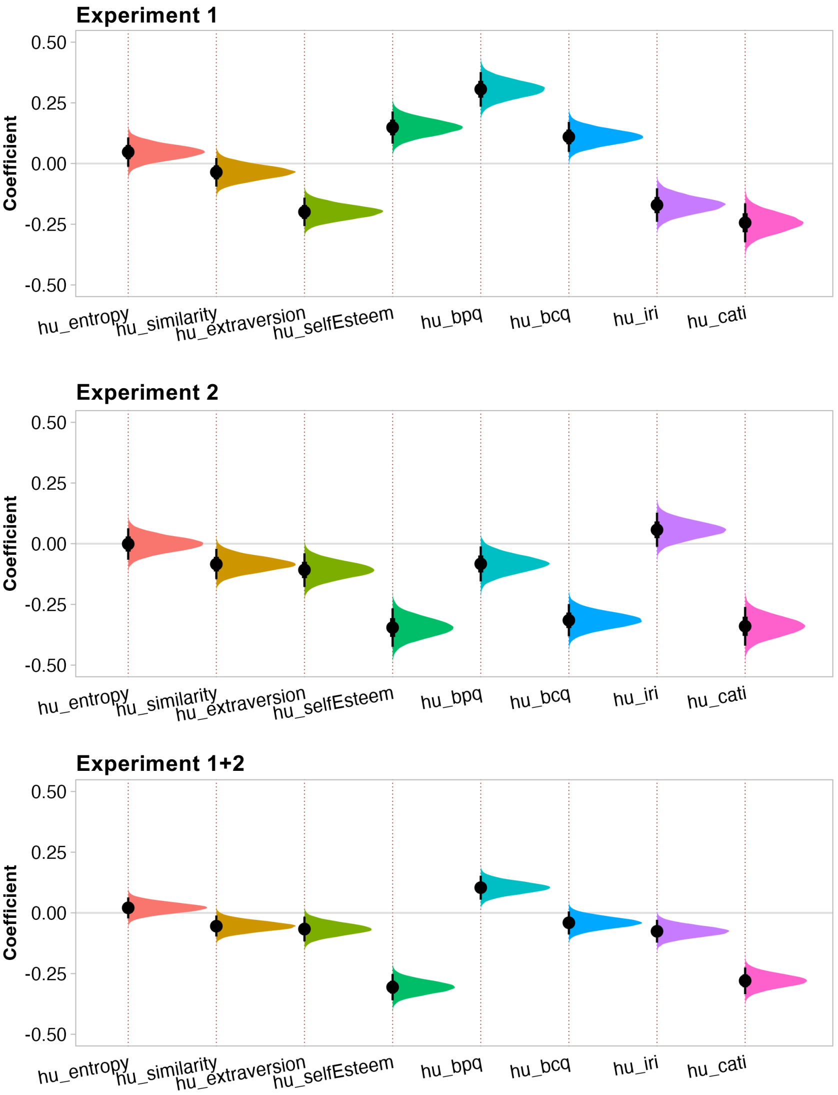

Supplementary Figure G) Influence of interindividual traits, movement similarity, and predictability (entropy) on enjoyment: Mu (enjoyment per se, i.e., rating between 11-100) parameter estimates from Exp1.17.

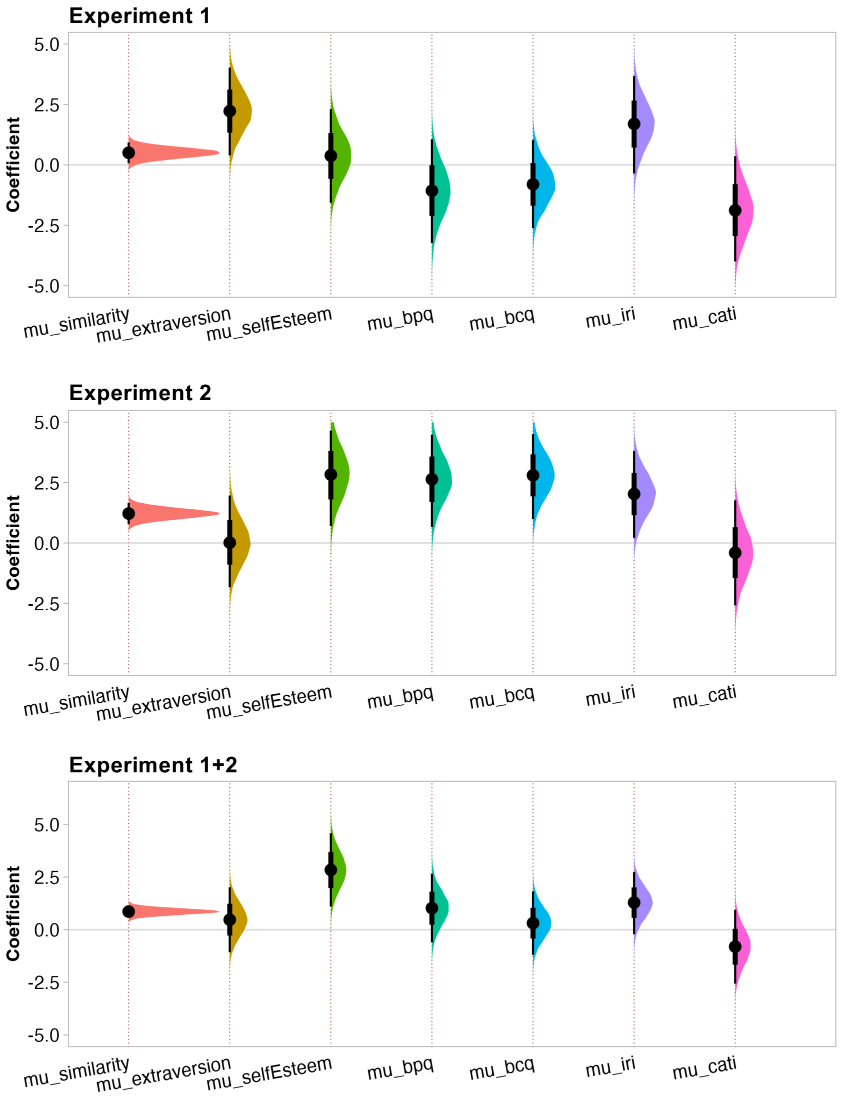

Supplement: Supplementary file 1 — Supplementary Information. [file 41598_2024_53191_MOESM1_ESM.pdf]
